# Editor’s Note: the project DanzArTe, a technology-based multicomponent dance movement

**DOI:** 10.1007/s40520-024-02754-2

**Published:** 2024-04-10

**Authors:** Nicola Veronese

**Affiliations:** Department of Internal Medicine and Geriatrics, University of Palermo, Via del Vespro, 141, Palermo, 90127 Italy

Recently, Barbagelata et al. reported the experience of the project entitled DanzArTe [1]. In this nice study, the Authors explored the efficacy of a technology-based multicomponent dance movement intervention among community-dwelling and nursing home older adults to improve several outcomes, including resilience. In my opinion, this study explores several important “hot” topics in geriatric medicine, namely the use of technology, the inclusion of frail people in intervention studies and the consideration of resilience and quality of life/satisfaction among clinical outcomes of interest [1]. 

As our global population ages, promoting the well-being and resilience of older adults has become an increasingly urgent priority. Aging is often accompanied by a myriad of challenges, including physical decline, cognitive impairment, social isolation, and emotional distress. In the face of these challenges, building resilience—the ability to adapt positively to adversity and maintain psychological well-being—is essential for aging successfully. Recognizing the importance of resilience in aging, the DanzArTe Emotional Well-being Technology Project seeks to empower older adults through a novel multicomponent intervention that integrates physical activity, cognitive training, and emotional expression.

At the heart of the DanzArTe project is the recognition of the interconnectedness of physical, cognitive, and emotional well-being in older adults. Physical activity has long been recognized as a cornerstone of healthy aging [2], with numerous studies demonstrating its benefits for physical health, cognitive function, and emotional well-being. By incorporating elements of dance therapy, the DanzArTe project not only promotes physical fitness but also provides a creative outlet for self-expression and emotional release, fostering a sense of joy, connection, and vitality among participants living in community or nursing home.

Furthermore, the cognitive training component of the intervention targets cognitive domains such as memory, attention, and executive function, which are essential for maintaining independence and quality of life in older adults [3]. Through engaging cognitive exercises and games, participants are challenged to sharpen their cognitive skills, enhance their problem-solving abilities, and build cognitive resilience against age-related decline. By stimulating cognitive function in conjunction with physical activity and emotional expression, the DanzArTe project aims to optimize brain health and cognitive vitality in older adults.

In addition to its physical and cognitive components, the DanzArTe project integrates innovative digital technologies to enhance the delivery and accessibility of the intervention. Mobile applications, virtual reality platforms, and wearable devices are utilized to deliver personalized exercise routines, track progress, and provide feedback to participants in real-time. These technologies not only facilitate remote participation and monitoring but also empower older adults to take an active role in managing their health and well-being. To improve technology awareness is one the main aims of the next few years, in my opinion: it was reported that over 60% of people between 55 and 64 years state that technologies’ costs are a barrier, whereas nearly half of those over 80 years state that a lack of knowledge is an important issue to use technologies [4]. 

In conclusion, the DanzArTe Emotional Well-being Technology Project exemplifies the transformative potential of multicomponent physical and cognitive interventions for promoting resilience and emotional well-being in older adults. By integrating evidence-based strategies from multiple disciplines and leveraging the power of technology, the project offers a holistic approach to aging that empowers older adults to thrive physically, cognitively, and emotionally. As we continue to explore innovative approaches to aging, let us embrace the spirit of resilience, creativity, and possibility embodied by the DanzArTe project, and together, build a future where all older adults can age with dignity, purpose, and joy.
